# Severe Sequelae to Mold-Related Illness as Demonstrated in Two Finnish Cohorts

**DOI:** 10.3389/fimmu.2017.00382

**Published:** 2017-04-03

**Authors:** Tamara Tuuminen, Kyösti Sakari Rinne

**Affiliations:** ^1^Medicum, Department of Bacteriology and Immunology, University of Helsinki, Helsinki, Finland; ^2^KristinaMedi Oy, Kauhajoki, Finland

**Keywords:** sick building syndrome, autoimmune conditions, malignancies, multiple chemical syndrome, environmental molds, hypothyroidism, indoor air, mold-related illness

## Abstract

The presence of toxic indoor molds with accompanying bacterial growth is clearly detrimental to human health. The pathophysiological and toxicological effects of toxins and structural components of molds and bacteria have been clarified in experiments conducted in tissue culture and animals, and there is convincing epidemiologic evidence; nonetheless their implications for human health are either ignored or denied, at least in Finland. In this communication, we describe two cohorts suffering severe sequelae to mold-related illness. One cohort is a nine-member family with pets that moved into a new house, which soon proved to be infested with pathogenic molds. The other cohort consists of 30 teachers and 50 students from a mold-infested school building. The first cohort experienced a plethora of mucosal irritation, neurological, skin, allergic, and other symptoms, with all family members ultimately developing a multiple chemical syndrome. In the second cohort, we detected a greatly elevated prevalence of autoimmune conditions and malignancies. We claim that mold-related illness exists in multiple facets; if not simply a transient mucosal irritation or even an increased risk of asthma onset or its exacerbation. We propose a scheme to explain the natural course of the mold-related illness. We recommend that future studies should combine data from, e.g., cancer, autoimmune, and endocrine disorder registers and neurological and mental health or neuropsychological registers with mold-exposed individuals being monitored for prolonged follow-up times.

## Background

**Figure 1:**
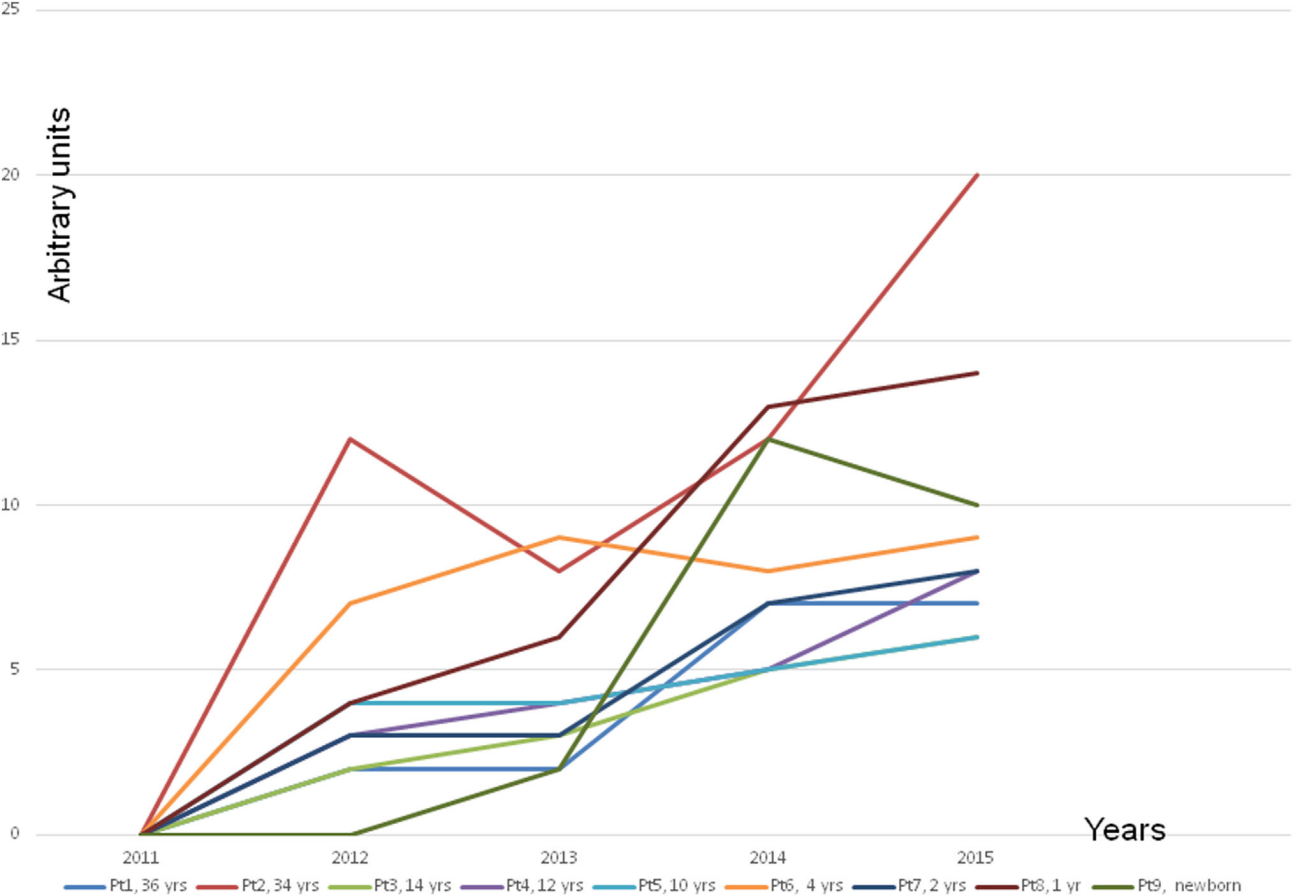
**The frequency of symptoms other than infections**. The other symptoms were insomnia, migraine, motor and sensory peripheral neuropathy, headache, tremor, twitching, tic of the body and in the eyes; tiredness, exhaustion; increased blood pressure, increased heart rate; muscle pain, joint pain; numbness in the hands and feet; weakness; feeling thirsty; feeling cold, shivers; balance problems; muscle weakness; slightly cyanotic limbs; irritability, melancholy, nervousness, memory disorders; facial flushing, skin rash, full-body rash, urticaria of the whole body; intestinal disorders, e.g., diarrhea or constipation; increased allergic reactions; excessive sweating or no sweat at all; crusted skin behind the ears or in the scalp of children; increased secretion of ear wax, jamming in matters in children, restlessness, difficulty falling asleep; retardation in growth; gastrointestinal reflux; too high or too low acidity of the stomach; weight loss without deliberate dieting; swelling of the face and the abdomen region; increased frequency of urination; nightly horror scenes in children; unexplained fever spikes; pallor, dark under the eyes; prolonged jaundice in a newborn; hypothermia; bedwetting in a child who was for many years dry at nights; child’s rage; jaundice of skin; night waking in children; significant hair thinning; dermatitis of the face skin and acne-like symptoms also in adults; unexplained vomiting in children; easy bruising; hypersensitivity to noise in children; dark urine in children although well hydrated.

The recent publication of the Audit Committee of the Finnish Parliament (1) indicated that approximately 7–9% of terraced houses; 6–9% of apartment buildings; 12–18% of schools and kindergartens; 20–26% of nursing homes, hospitals, and outpatient departments; and 2.5–5% of offices have been significantly damaged with dampness and are infested with indoor molds. It has been estimated that approximately 800,000 or every seventh Finnish citizen (1) has been exposed to some extent and become sensitized to compounds present in poor quality indoor air. However, since there is no ICD-10 coding system for mold-related illness, its exact incidence is unknown. If one extrapolates from the above presented figures (1), one could argue that the incidence of mold-related illness may be much higher than the incidences for cardiovascular conditions, cancers, and accident-induced traumas. Despite (or perhaps due to its ubiquitousness and its all-too-frequent involvement in highly publicized litigation issues) there is no consensus by the medical authorities on how this disease should be recognized. Marginalization of patients (2) with this disorder results inevitably in serious social welfare problems. The very recently (11/2016) issued, and in our opinion totally biased, Current Care Recommendations for treating patients suffering from moisture-damaged buildings (3) only aggravate this injustice. These “recommendations” are inconsistent with the constitutional rights guaranteeing to all Finnish citizens that *“Anyone who cannot obtain decent livelihood has a right to receive appropriate subsistence and care”* (4). The official rhetoric of denial of the mold-related illness (5–7) can be summarized into three main points: (1) asthma is the only clear disease that can be associated with moisture-damaged buildings; (2) there is not sufficient evidence that dampness and mold overgrowth are associated with adverse health conditions; and (3) the mechanisms causing dampness-related illness are still unknown.

In this publication, we describe two Finnish cohorts; our goal is to raise awareness that indoor dampness associated with toxic molds and bacteria overgrowth can cause a plethora of serious sequelae including neurological, autoimmune diseases, e.g., hypothyroidism as well as cancer and multiple chemical syndrome (MCS), and even higher mortality.

The term multiple chemical syndrome has been defined (8–11); it is already accepted as a distinct clinical entity in several EU countries. The criteria for MCS definition have been set (8, 9) as (1) the condition is chronic; (2) with symptoms recur reproducibly; (3) in multiple organ systems; (4) in response to low levels of exposure; (5) to multiple unrelated chemicals and which; (6) improve or are resolved when incitants are removed. Although mold-related illness has not been associated with the development of MCS, we will convincingly demonstrate that MCS illness can indeed be a consequence of mold-related disease when the exposure to toxic molds has been prolonged and the symptoms have become chronic.

There have been suspicions that mold-exposed individuals experience a higher prevalence of hypothyroidism, and therefore we started to collect evidence to investigate this association. We reviewed the medical records of the personnel in one school, which had been identified as a mold-infected building. We will present novel data that the presence of toxic molds in a building may indeed associate with a higher prevalence of hypothyroidism in its users/inhabitants than in the general population. This prevalence was calculated using statistics of thyroid hormone substitution therapy in 2015 provided by the Social Insurance Institute of Finland, KELA. We document also that mold-related disease can include malignancies. The incidences of breast cancer and lymphomas were calculated in the personnel from the school and compared to the register data detailing the corresponding incidence of the region (9). The overall prevalence of autoimmune disease was compared to our best estimate of the corresponding value in the general Finnish population.

The evidence that there is an association for the development of malignancies in individuals chronically exposed to toxic molds is an even more challenging task because this requires a lengthy follow-up and access to several cancer registers. In this report, we present data that should raise concern about the potential carcinogenic properties of mycotoxins and other toxic products present in moldy buildings.

### Ethics Statement

This research is IRB exempt because it is a retrospective chart review study. Informed written consent was obtained from the mother (Cohort 1) and all the patients (Cohort 2) whose medical records were reviewed.

### Cohort 1

The evidence that toxic indoor molds can cause chronic respiratory symptoms, cognitive disorders, neurological symptoms such as insomnia and migraine, failure to thrive in a newborn, and MCS in occupants of a moisture-damaged house was collected by careful inspection of medical histories and interviews of the affected individuals.

A family of nine with seven children, three cats, and two dogs moved into a brand new house in November 2011. The seventh child was born in this problem house in November 2013. None of the family members had required any long-term medication prior to November 2011. During their infancy, two of the family’s daughters had suffered from milk or soy allergy, but these symptoms declined as the girls grew older such that before their move into the new house, they were asymptomatic. The mother had been sensitized to molds in her workplaces during the years 2006–2009 and 2010–2011. At that time, she experienced nose and ear itchiness, which later changed to ear infections and respiratory symptoms, but by November 2011 she was asymptomatic because she changed her place of employment. Soon after moving into their new house, the parents smelled a strong odor of sewage, which the building contractors attributed to inadequate ventilation. The contractors made several attempts to correct the defect in the sewerage; however, the odor remained.

Approximately 1 month later, all members of the family were experiencing many symptoms, e.g., intense mucosal irritation of the eyes, coughing, pain in the throat region, throat infections, shortness of breath, sinus infections, congestion, etc. These symptoms usually appeared whenever they were present in the house. These mucous membrane irritations led to a cycle of infections, which resulted in many medical consultations. Soon afterward, the symptoms experienced by the family members also developed in various organs (Figure 1). Many had headaches, all of them experienced some kind of skin symptoms, 6/9 had functional abdominal symptoms, at least 3/9 had either fever or low body temperature, and at least 4/9 suffered muscle and joint pains. Four of the children developed asthma, which required medical treatment. With time, seven members had developed food and pollen allergies. It is significant that the mother’s aunt, who stayed in the house between October and November 2013, suffered a migraine attack sufficiently severe to require hospital care. She had also experienced severe cough during her stay in the house.

Even the family’s pets became unwell; the dogs had throat infections, cats had “flu” and eye infections, one of the cats displayed asthma-like symptoms, especially in spring and autumn 2011–2013 (in 2013, the pets were given away).

The family’s seventh child was born in the problem house and suffered from a prolonged bout of jaundice (3 months), his excrements were strangely dark, and had a horrible smell instead of the usual milky feces. He failed to thrive during the first few months.

**Figure 2:**
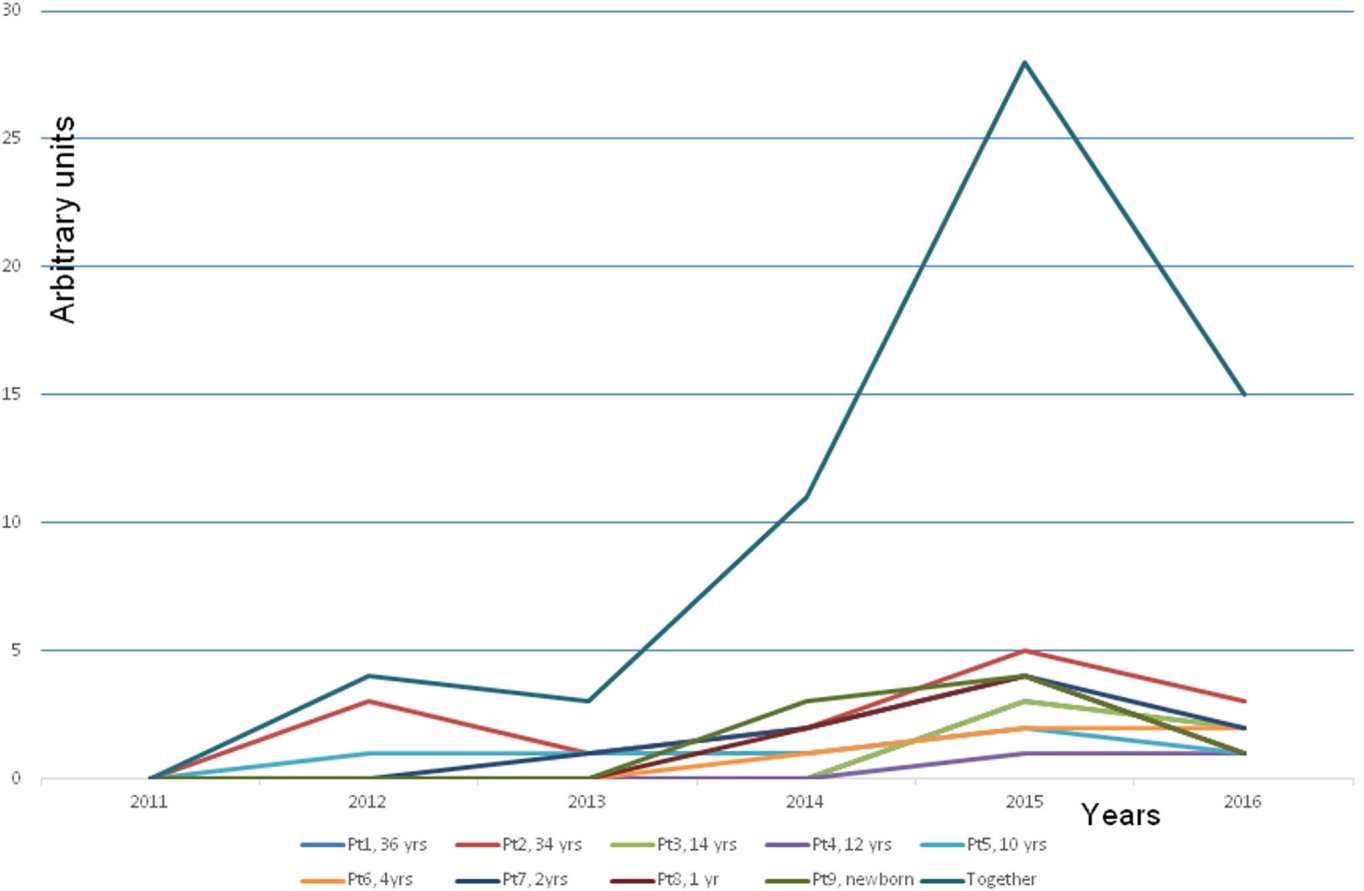
**Development of a multiple chemical syndrome (MCS illness) in every family member**. Note that the symptoms developed gradually along with increased time of staying in the problem house. The presentation of the MCS illness was estimated by an arbitrary score. The blue line is the cumulative score of all the members. *The explanation of the arbitrary score*: +, hypersensitivity to strong detergents, softeners, and perfumed hygiene products. A child reacts to the odor by avoiding the perfume or the smell of a person or an object. ++, a clear hypersensitivity to detergents and perfumed products. A child avoids entering several departments in a department store with a strong smell burden (e.g., bags, shoes, textiles, toys, and detergents) and avoids contacts with people who have strong smell from their clothing, etc. +++, a clear hypersensitivity to perfumes and flagrancies, the same as above but in addition hypersensitivity to gasoline, exhaust, and windshield washing fluid, new fabrics, and new furniture. ++++, reacts strongly by a production of mucous excreta and itching feeling on the mucosa. The symptoms as above but in addition the hypersensitivity to adhesive surfaces, printing ink, plastic, rubber, markers, ballpoint pens, new toys, books, games, and textiles. Symptoms appear also in various dusty environment, e.g., to road dust. +++++, as above but in addition the hypersensitivity to perfume-free detergents and even to natural scents of flowers, trees, grass, soil, etc.

The next dramatic turn experienced by all family members was the development of MCS or MCS illness as it is alternatively described. The severity of the disease was variable (Figure 2). In Finland, there is no recognition of the MCS, only the national R68.81 code, which according to official policy is nihilistically called “a feature,” not a disease. It is noteworthy that the children even reacted to new toys, which are clearly at odds with the officially propagated explanation that MCS illness is a conditional reflex (12).

In 2015, the presence of *Penicillium* and *Aspergillus* was confirmed. For the latter species, the cell equivalent (CE, which is defined as any particle of mold or spore containing DNA) value was 13,000 CE/g, compared to the maximum permitted cutoff value of 2,400 CE/g. Furthermore, various bacterial species were also detected from the material taken from internal surfaces in the majority of the living space in quantities greatly exceeding the reference values. The family moved to a rented apartment in February 2016, and gradually their symptoms have started to resolve.

### Cohort 2

Our evidence of a cluster of very rare diseases, a high incidence of oncologic diseases, and autoimmune conditions associated with a moisture-damaged school has been obtained through a lengthy, personally conducted follow-up of all the patients described below.

A wooden school was built in a small city in the late 1880s. Over the years, this building has undergone several reconstructions. During the last 20 years, there have been approximately 30 occupants working in this building for variable periods of time (3–20 years; min–max). Medical records collection at the school was supervised by a medical doctor employed at the school since 1985. The records reveal an astonishingly high incidence of severe morbidity and a high mortality rate associated with even a short period of working or studying in this building (median 12 years). All of the available demographic and clinical data collated from the medical records are presented in Table 1.

**Table 1:** **The morbidities and mortalities diagnosed in a personnel working in the problem school (Cohort 2)**.

Person	Years of exposure	Gender	Profession	Morbidities	Employment status
A	25–30	Male	School rector	Inclusion body myositis	Retired due to age
				Exacerbation of allergy	
				Irritation of mucosa	
				Voice problems	

B	20	Female	Instructor	Asthma	Partly employed
				Sjögren syndrome	
				Thyroiditis	
				Dystonia	
				Migraine	

C	12	Male	Instructor	Vasculitis with purpura	Working

D	13	Female	Instructor	Goiter with hypothyroidism	Working
				Diabetes	
				Sleep apnea	
				Skin symptoms	

E	5	Female	Instructor	Chronic flu-like illness	Working half time
				Cough	
				Sneezing	
				Voice problems	
				Eye irritation that led to iritis	

F	20	Female	Special teacher	Breast cancer	Retired due to age

G	12	Female	Teacher	Asthma exacerbation	Retired due to disability
				Chronic sinusitis	
				Chronic otitis	
				Allergy	
				Eosinophilia	
				Hypothyroidism	
				Nasal polyps (st post polypectomy)	
				Difficulties to concentrate	
				Memory problems	
				Chronic fatigue	
				Depression	
				Altogether, history of sickness that lasted for 35 years	

H	20	Female	Special teacher	Breast cancer	Working

I	5	Female	Instructor	Chronic sinusitis	Working
				Allergic rhinitis	
				Partial hearing loss	

J	10	Female	Special teacher	Neurosarcoidosis	To be retired due to disability
				Hypothyroidism (severe)	
				Intestinal stoma	

K	3	Female	Instructor	Chronic sinusitis	Working
				Chronic otitis	
				Chronic eye irritation	
				Joint pains	

L	20	Female	Instructor	Asthma	Retired due to age
				Allergy	
				Breast cancer	

M	5	Female	Special teacher	Underlying disease:	Retired due to disability
				Cilium dysfunction
				The following symptoms exacerbated:
				Chronic sinusitis
				Bronchiectasias
				Chronic otitis
				Pneumonia
				Joint pains
				Fybromyalgy
				Asthma
				Hypothyroidism
				In the beginning during the holiday period, the symptoms relieved, but later they became chronic, and now her morbidity is severe

N	8	Female	Instructor	Non-smoker, previously healthy	Dead
				Chronic rhinitis	
				Flu-like symptoms	
				Chronic bronchitis	
				After the prolonged period of flu illness died from sepsis	

O	10	Female	Special teacher	Lung cancer (non-smoker)	Dead

P	10	Female	Instructor	Asthma	Partly employed
				Chronic sinusitis	
				Multiple chemical syndrome illness	
				Severe sleep apnea	
				Memory problems	
				At the time of this communication her thyroid function and neurological disorders are being investigated	
				High sensitivity to poor indoor air	

R	10	Female	Instructor	Chronic sinusitis	Working
				Psoriasis-like skin problems	
				Eye irritation	
				Chronic otitis	
				Voice problems	
				Memory problems	
				Problems to concentrate	
				Chronic fatigue that led to depression	

S	4	Female	Special teacher	Goiter	Retired due to age

*To make the assessment of the disease category more illustrative we use colors: blue for autoimmune diseases; violet for oncology; red for nervous disorder; brown for asthma, and green for upper and lower respiratory symptoms, eye and skin irritation*.

In addition to individuals suffering from serious symptoms (Table 1), many other employees suffered from chronic eye and ear irritation, sinusitis, bronchitis, fever, skin problems, fatigue, and joint pains, and some experienced exacerbation of some underlying disease. Some occupants, whose exposure to the school’s poor indoor air was short, became asymptomatic when they moved to another school that did not seem to have any indoor air problems. For those whose symptoms and illnesses became chronic, the median exposure time was approximately 12 years. The majority of students in the school experienced “flu-like” symptoms and fatigue.

Table 1 reveals extremely alarming statistics: 2 out of 30 occupants developed very rare autoimmune diseases: 1 teacher suffered from inclusion body myositis (the average incidence in Finland is 1:1,000,000), and the other suffered neurosarcoidosis (the average incidence in Finland is 1:500,000). Altogether, more than every third building occupant (11/30 = 36.6%) experienced different types of autoimmune conditions. Depending on how one calculates the value, the average prevalence of autoimmune diseases in Finland is in a range of 5–8%, i.e., the observed prevalence is at least fourfold higher than the average. Hypothyroidism or goiter was diagnosed in every fifth occupant (6/30 = 20%), whereas in Finland, the average calculated prevalence is known to be 5.78%. Therefore, in our cohort the prevalence of thyroid dysfunction was elevated by 3.4-fold.

The incidence of oncologic diseases was also greatly elevated; lymphoma was diagnosed in 2 out of 50 students. One student had attended the school for 1.5 years and the other for 4.5 years. Therefore, the calculated incidence is 666:100,000 (which is not an exact estimate due to the small size of the cohort). From the register of lymphomas for the same region, we estimate an average incidence of 14:100,000. Therefore, in our small cohort, the incidence was 47.5-fold higher. We documented 3 breast cancers among 25 female teachers who worked in the school during the 20-year observation period. The calculated incidence is therefore 600:100,000, whereas the average incidence for the region is 101.5:100,000. Thus, the incidence of breast cancer in our cohort was elevated by approximately sixfold. In addition, we documented also an extremely high mortality rate among what should be a potentially healthy population (i.e., schoolchildren and teachers) during the period 1980–2011: one student died from pneumonia; two students died from lymphoma; one non-smoker teacher died from lung cancer; and one young and previously healthy female instructor succumbed to sepsis, which is very rare in Finland.

From Table 1, we can also see that 2 Teachers retired prematurely since they had been diagnosed with a work disability; 2 teachers were forced to work only part time because of their disability. In general, the employees of the school were frequently absent from work due to sickness. High employee morbidity is expensive, i.e., each day of sick leave costs approximately 250–300€.

Microbiological investigations performed in 2011 revealed an excessive growth of *Paecilomyces, Exophiala, Penicillium, Aspergillus peniccilloides/restrictus, Aspergillus fumigates, Tritirachium*, and Paecilomces species (all above the cutoff values of 96–194 cfu/g) in mineral wool and other types of insulation material. After appraisal of these microbiological data, this school was closed, but it was reopened again as a primary school without adequate consultations with the community. Subsequently, it was closed again according to our (KR) recommendations.

### The Buildings

In both cohorts, the evidence that the microbiota was related to moisture in the building was substantiated by indoor air studies and validated culture techniques performed by certified environmental experts.

## Discussion

Here, we present convincing evidence that toxic indoor molds can indeed cause not only chronic respiratory symptoms or irritation of mucous membranes but also cognitive and neurological disorders such as insomnia, migraine, motor, and sensory peripheral neuropathy; failure to thrive in a newborn baby. Furthermore, all occupants of the moisture-damaged house suffered from MCS (Cohort 1). We claim that toxic molds can cause severe morbidity and mortality in adults and children, even domestic pets; and a relatively short stay in a damaged building is a potential hazard to health and life. We also present evidence of a cluster of very rare autoimmune and oncologic diseases associated with a moisture-damaged school. For example, in Cohort 2 we documented that 36.6% of the teachers suffered from autoimmune condition, whereas in the general population the prevalence is much lower—approximately 5–8%—depending on which source is used in the calculation. The incidence of cancer was also alarmingly high. These diseases can be hardly overrepresented by chance. Although some may be skeptical of our data, we can confirm that both of the buildings were mold infested as proven by certified professional bodies, and that the clinical data were collected by careful evaluation of the medical records.

As far as we are aware, this is the first time that MCS illness has been demonstrated to occur after prolonged exposure to indoor molds when the disease turns chronic. This conclusion is justified when we consider the symptoms experienced by all the occupants investigated in Cohort 1 (Figure 2). Even very small children whose reactions cannot possibly be attributed to conditioning or an exaggerated fear of putative environmental threats (i.e., young children are always eager to play with new toys and are not old enough to understand the concept of an environmental threat) developed nausea, headaches, and respiratory symptoms when they were confronted with strong perfumes or detergents from new toys, textiles, and furniture. Unlike the situation in some other EU countries, regrettably in Finland MCS illness has not yet gained any official recognition. Instead, individuals suffering from this disorder are referred to psychiatrists, their symptoms labeled as psychosomatic, they themselves may be considered psychotic. All of the cases from Cohort 1 were not in need of psychiatric help; they had truly developed a MCS illness related to indoor molds (13).

Mold-related illness should not be viewed as a so-called medically unexplained syndrome, as has been claimed (14, 15). In our opinion, providing these patients with cognitive or behavioral therapy (14–16) is medically unethical—it represents a denial that mold-exposed individuals are suffering from a somatic illness. Moreover, cognitive/behavioral therapy is not effective (14). We can assume that providing the mold-exposed patient with only psychotherapy (14, 15) in combination with high dosages of corticosteroids while he/she continues to live or work in a hazardous environment is inappropriate “medication”; in fact, it will aggravate their risks of suffering severe morbidity and even dying. On the basis of the present data, we think that it is irresponsible to claim that indoor molds cause only transient irritation symptoms and pose only a 1.5-fold risk for the development of asthma (3, 5–7). Even though more and more knowledge is available on the mechanisms underpinning the health hazards associated with moldy environments, mold-related disease is still called a “non-disease,” or “somatoform disorder,” with some physicians trying to label it as a “fashionable” disorder, or stating that its sufferers are exhibiting hysteria (17). Mold-related illness is a somatic disorder; the symptoms are physical, not psychosocial problems, although this has been claimed for almost 20 years (17). In most cases, later it can become a psychosocial problem as patients suffer mental distress from their failure to convince physicians that they are ill. Our data show that occupying an infested building for even 2–3 years (either a home or a school) can seriously impair the well-being of potentially healthy individuals, even to the extent of loss of life. Therefore, any attempt by governmental/medical authorities to deny the serious effects of toxic molds on human health should be combatted.

Mold toxins may impair the immune system or other organs in many ways (18–29). They can either cause an overwhelming immune activation (autoimmunity) or an immune deficiency (e.g., leading to an inability to combat clones of malignant cells) making the individual liable to suffer infections that may be fatal, such as in our sepsis-induced death of a young, previously healthy, female instructor. Mold toxins and structural components of bacteria and fungi present in moisture-damaged buildings can exert synergic pro-inflammatory interactions (20) and trigger cellular autophagocytosis (25). Some peptide toxins from moisture-damaged surfaces may trigger immunotoxic and exert growth inhibitory effects in mammalian cells (19–21, 23). The anti-immune strategies mounted by pathogenic fungi including species also detected in moisture-damaged buildings have been described in a recent comprehensive review (24). The fungi have developed many complex strategies to evade attack by the host’s immune systems. One example of this complexity is the fact that some components of the molds and bacteria can activate a structure called the inflammasome (i.e., a pro-inflammatory action), whereas other components may hamper immune cell activation or even destroy immune competent cells, such as NK or T lymphocytes (21). It has been shown that *Aspergillus* species can inhibit the function of dendritic cells (25); these are crucial cells in immune defense as they first recognize and then present foreign molecules to the host’s secondary immune defense system. This is simply one of the many mechanisms of action of mold toxins; it is not the aim of the present communication to systematically review all the possible pathophysiological pathways. Instead, our goal is to highlight the evidence that toxic molds can be responsible for serious morbidity, even mortality. One characteristic feature of the mold-related illness is severe fatigue; this has been attributed to a toxicosis resulting from mitochondrial deprivation and low energy production (21).

The very high prevalence of hypothyroidism in mold-related illness is evident when reviewing the data from Cohort 2. The thyroid, the pancreas, and the heart are organs that require high energy production in order to function properly (28). Mycotoxins are cytotoxic and disrupt mitochondrial enzymatic functions, depriving tissues of energy (21). Thus, it is not illogical to argue that mold toxins would impair the metabolic activity of the thyroid gland leading to hypothyroidism.

A worrying signal emerges from the data from Cohort 2, i.e., a building with toxic mold overgrowth can cause a higher prevalence of malignancies. We appreciate that actually proving this association is a very challenging task. Nonetheless, our report has revealed a cluster of malignancies with a much higher prevalence than the prevalence of these malignancies in the general population. This association should be taken seriously. For example, although nowadays no one doubts the link between tobacco smoking and lung cancer, it should be recalled that it took several decades before this association was accepted by the medical profession. The recognition of the asbestos hazards also took a long time. Today, we should be concerned about the possible carcinogenic effects of indoor mold toxins and structural components hosting toxic microbes, especially compounds derived from materials pretreated with putative anti-mold substances (29). Cytotoxic and modulatory effects of mycotoxins on human breast cells have been recently documented (27).

The limitation of our study is that we present descriptive evidence based on the observation of only two cohorts. The strength of our publication is its novelty; we hope that it will encourage a change in attitudes toward patients with mold-related disorders. We also hope that it may initiate extensive, well-designed studies combining lengthy follow-up with data extracted from various disease registers.

**Figure 3:**
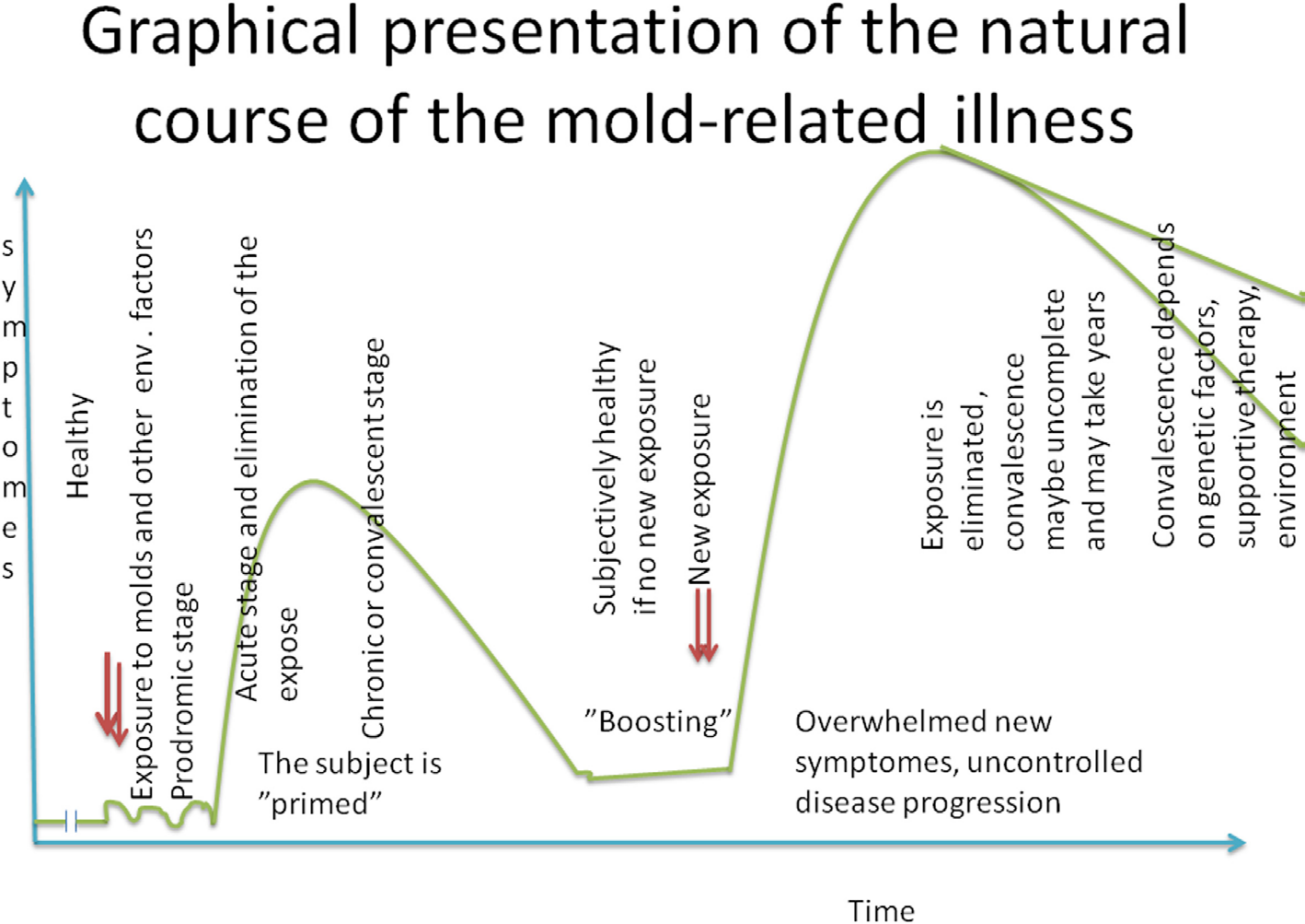
**Graphical presentation of the natural course of the mold-related illness**.

On the basis of our experience, we will present an empiric graphical reconstruction of the natural course of the mold-related illness (Figure 3). We believe that once a person has become sensitized to indoor molds, he/she will be sensitized for the rest of their life span. However, a subjectively asymptomatic healthy state can be achieved but that requires the avoidance of indoor molds, an appropriately controlled detoxification strategy, and a restoration of immune homeostasis with well-designed dietary interventions and other supportive therapies (30). If the patient becomes exposed again to mold-infested indoor air impurities, his/her symptoms may reappear almost immediately. The defense symptoms can be overwhelmed, and the disease may manifest itself with new symptoms, such as autoimmune diseases, neurologic disorders, etc. (Figure 3). A full recovery after the first encounter with mold-related toxins may take some time, but if these encounters continue for a prolonged period of time, full recovery may be unlikely; the disease can become chronic and spread to new organs.

## Concluding Remarks

In conclusion, we present clinical evidence that poor indoor air due to mold infestation can cause severe morbidity not restricted to asthma. These sequelae are oncological (31), neurological, autoimmune diseases, and even death. We emphasize that scientific discussion based on facts should be pursued without intervention from biased “opinion leaders.” What is not yet known should be studied with an open mind. In conclusion, the absence of the evidence should not be construed as evidence of absence.

## Author Contributions

TT and KR collected data and wrote the article together.

## Conflict of Interest Statement

The authors declare that the research was conducted in the absence of any commercial or financial relationships that could be construed as a potential conflict of interest.
